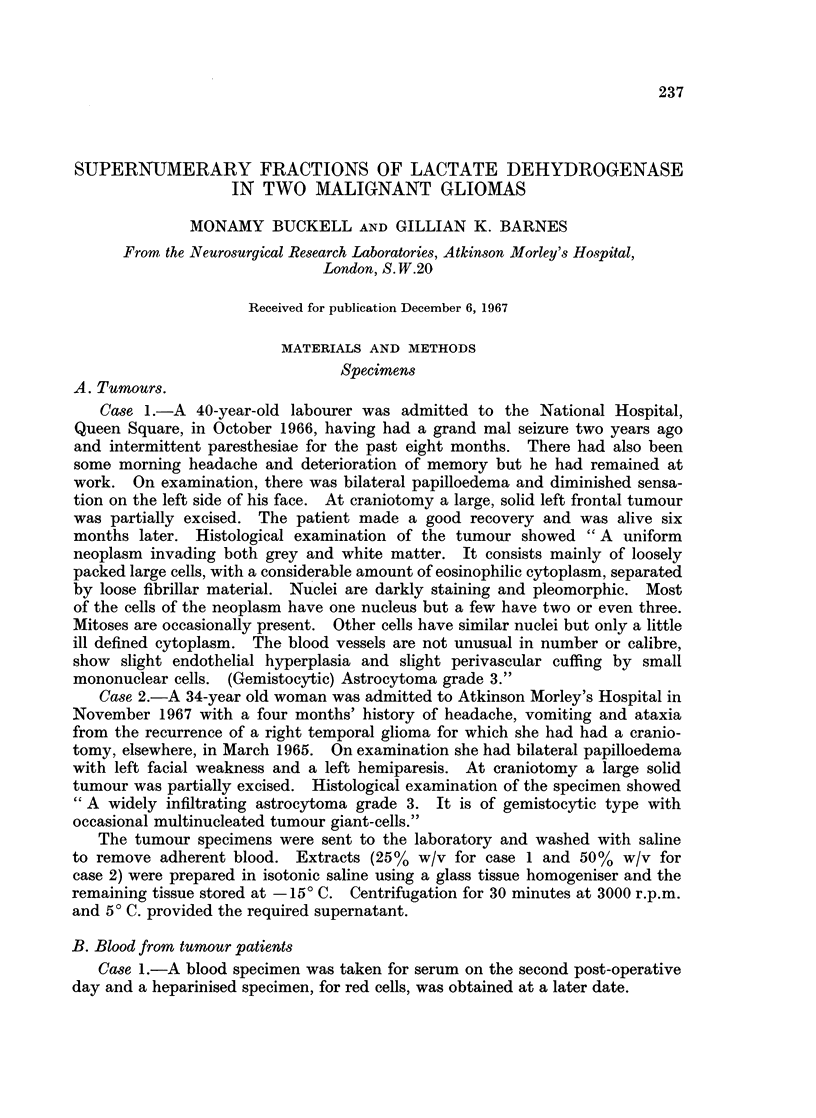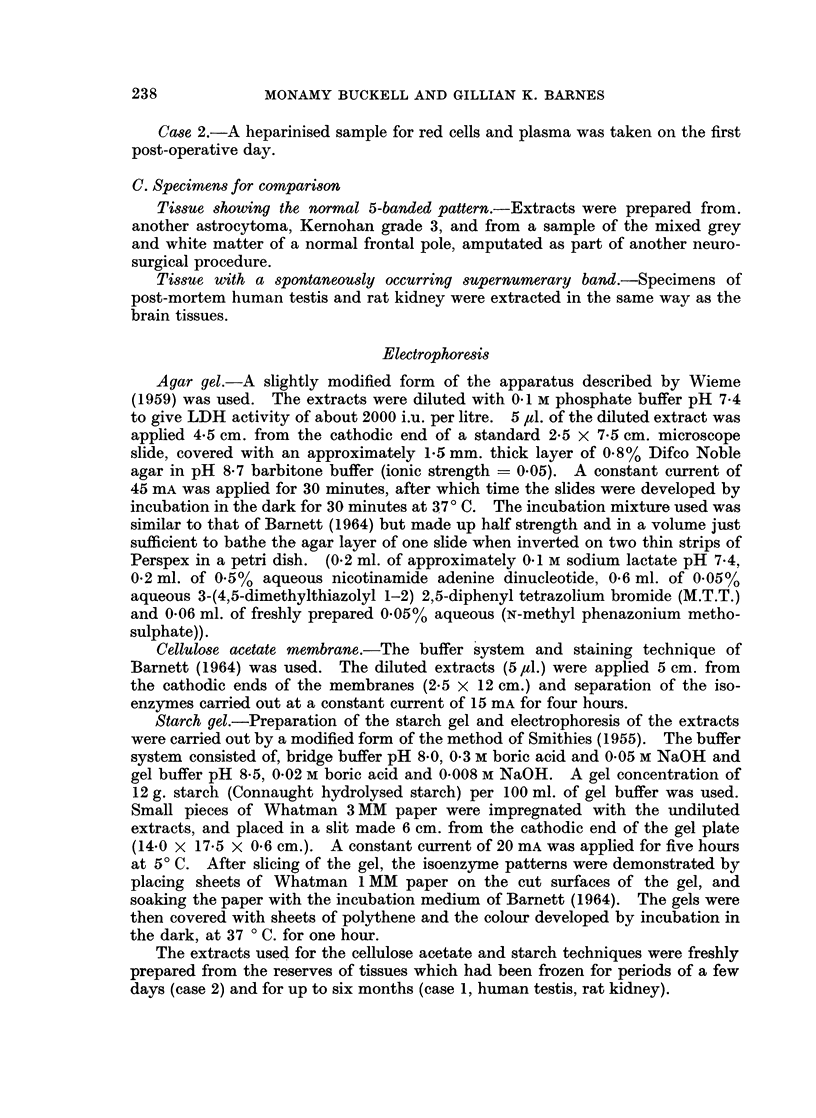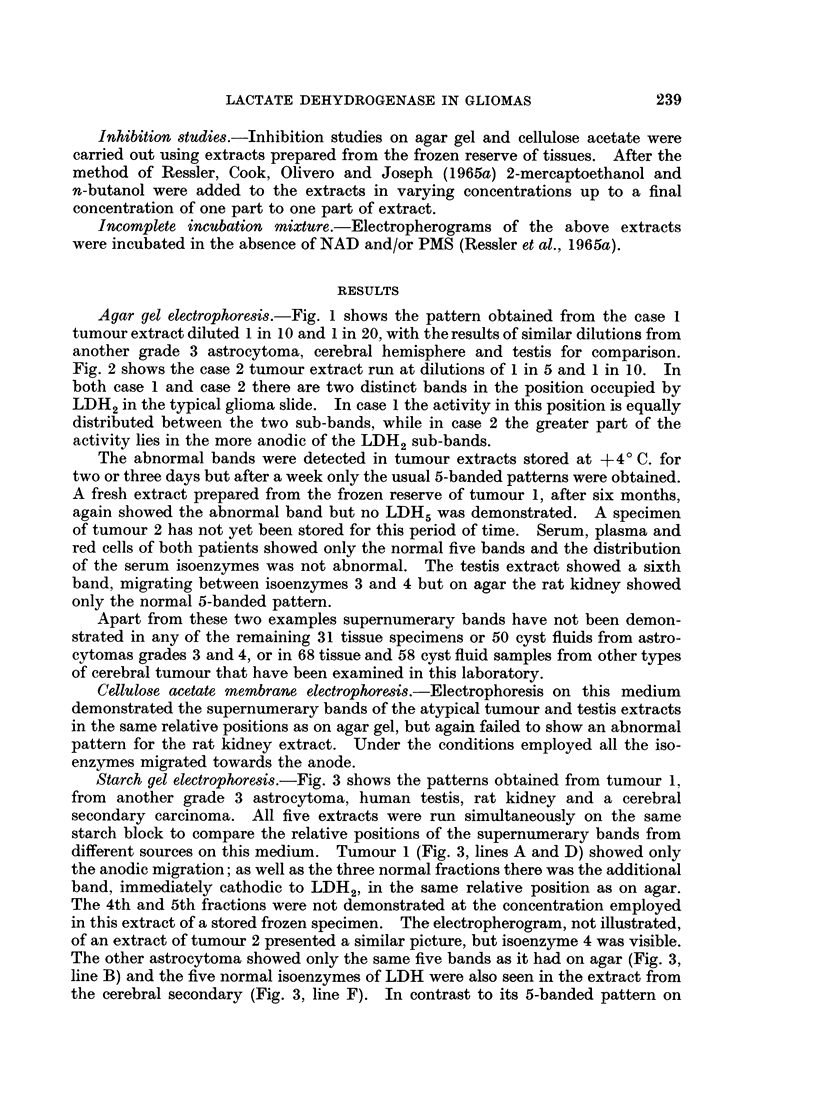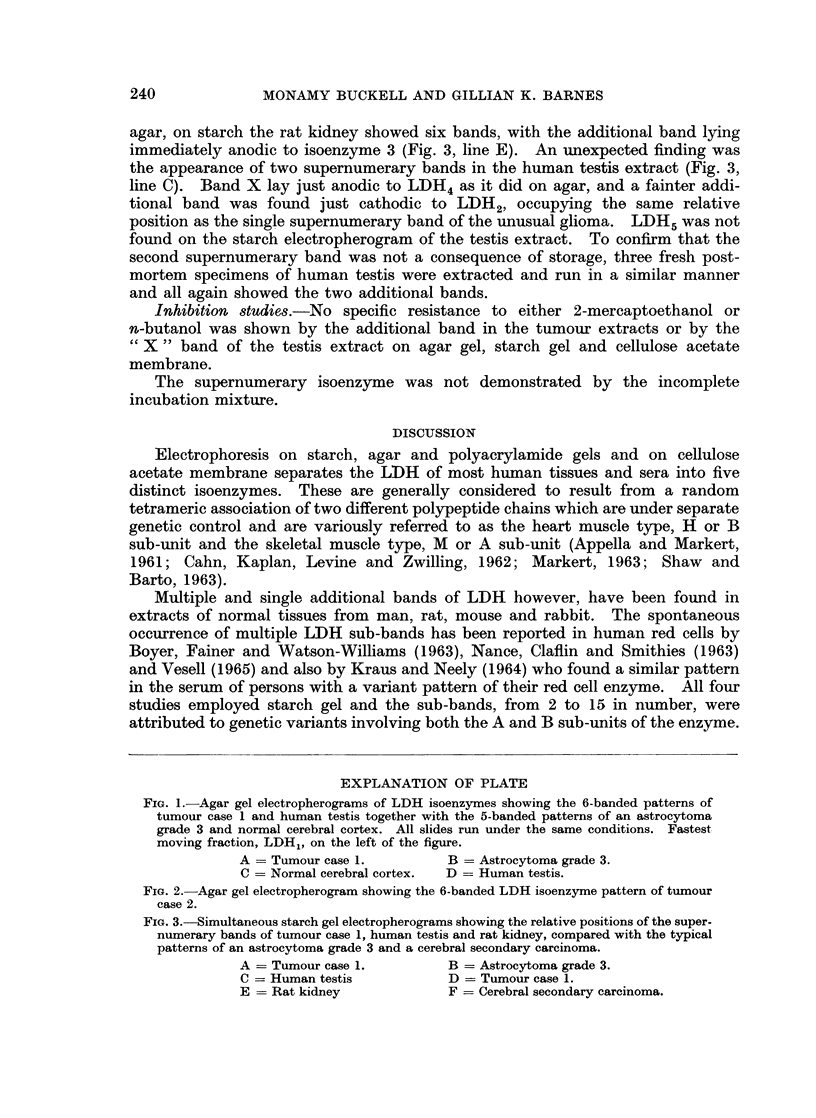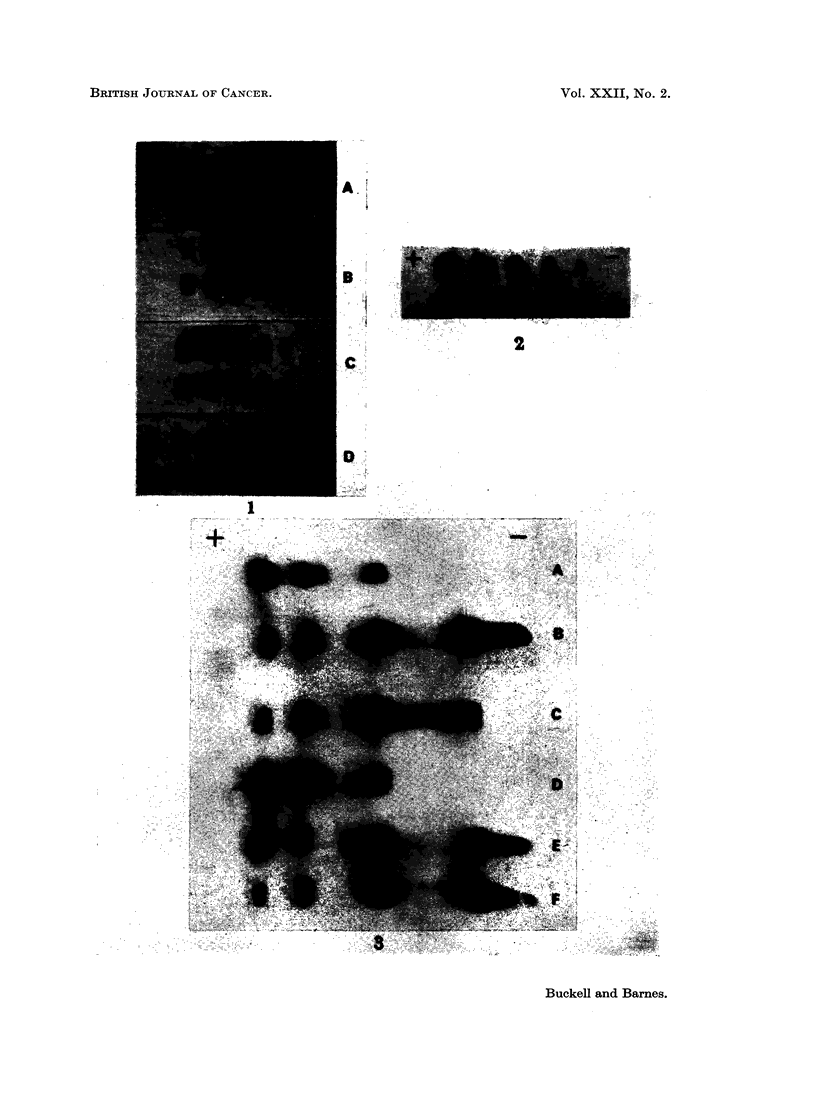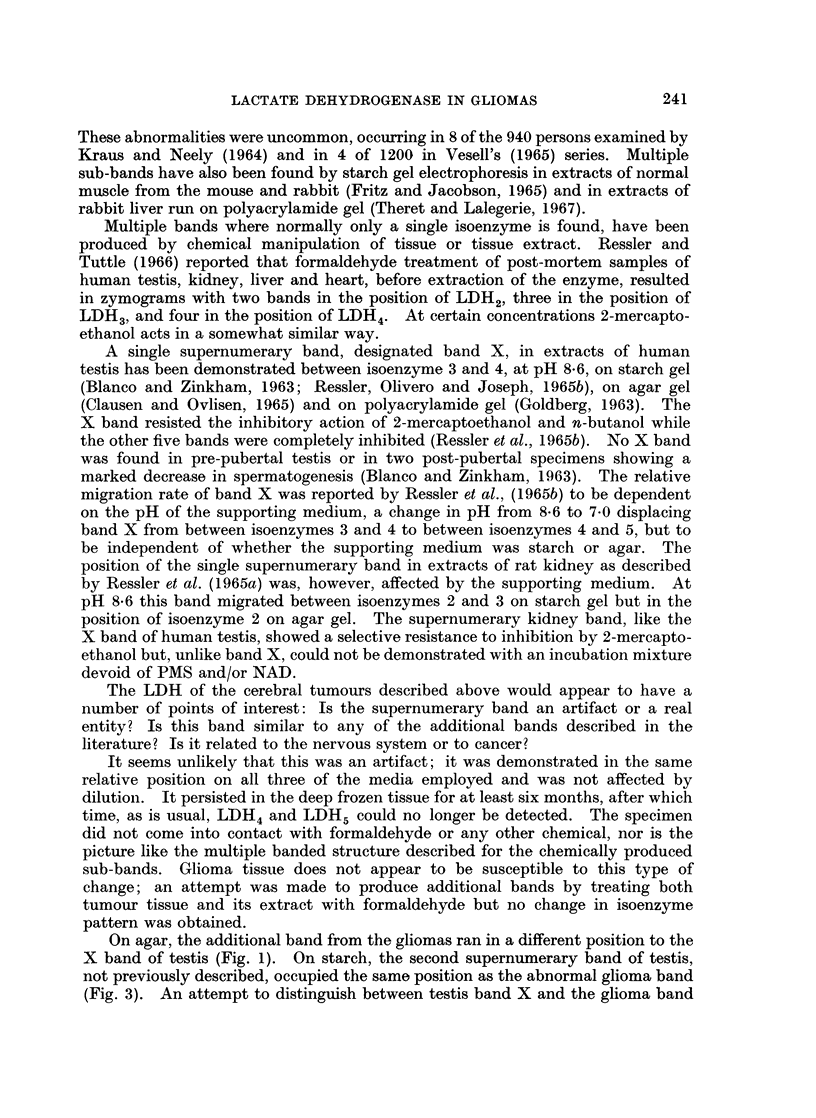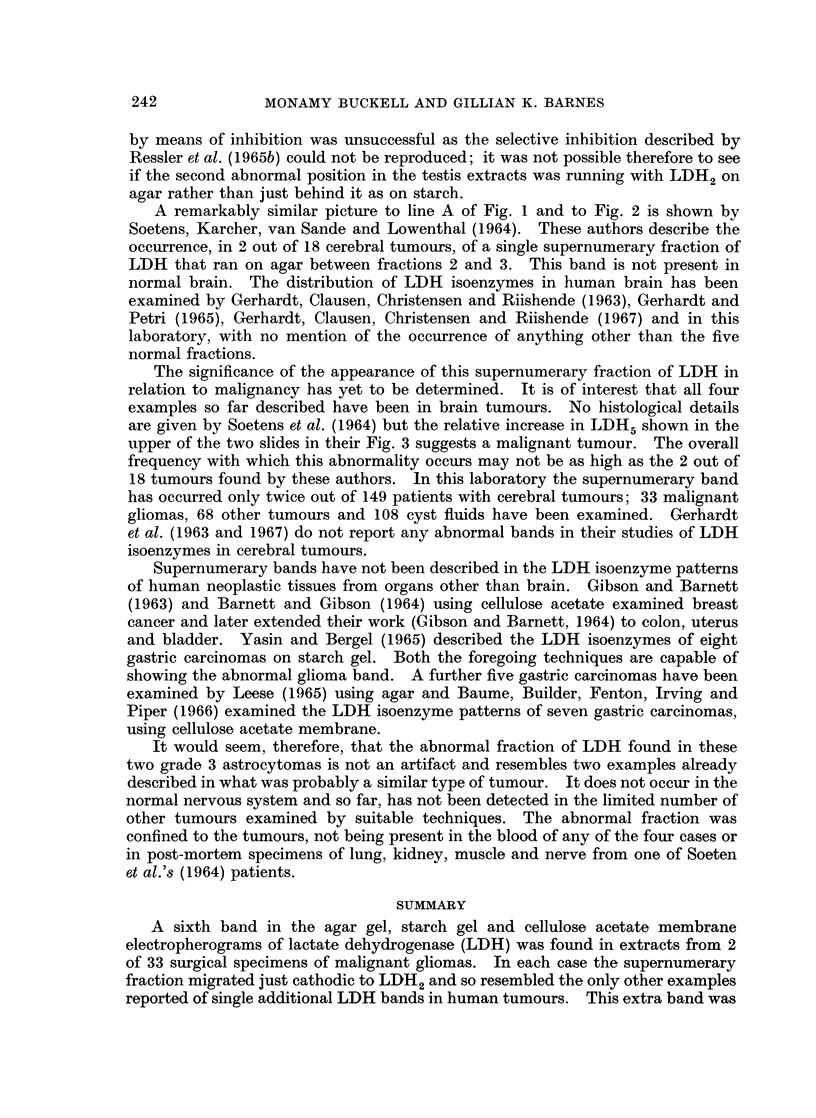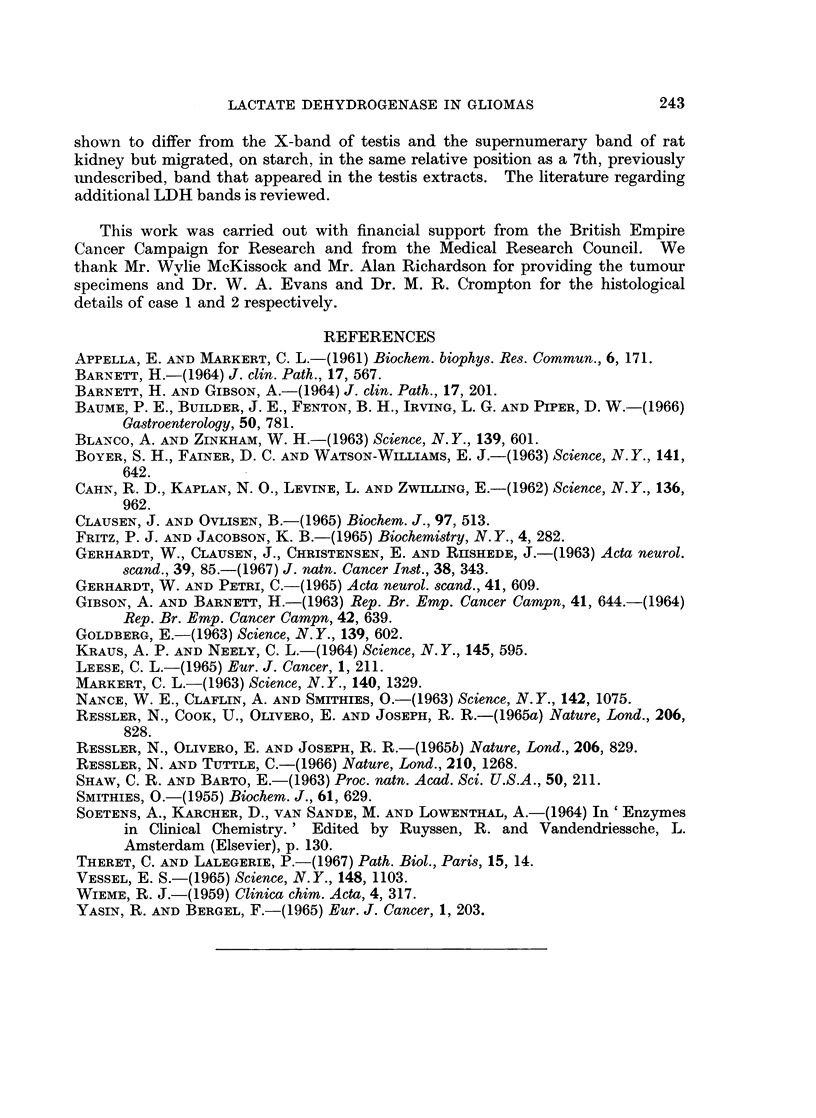# Supernumerary fractions of lactate dehydrogenase in two malignant gliomas.

**DOI:** 10.1038/bjc.1968.30

**Published:** 1968-06

**Authors:** M. Buckell, G. K. Barnes

## Abstract

**Images:**


### SUPERNUMERARY FRACTIONS OF LACTATE DEHYDROGENASE IN TWO MALIGNANT GLIOMAS

MONAMY BUCKELL AND GILLIAN K. BARNES

*From the Neurosurgical Research Laboratories, Atkinson Morley's Hospital, London, S.W.20*

Received for publication December 6, 1967

### MATERIALS AND METHODS

### *Specimens*

*A. Tumours.*

*Case* 1.—A 40-year-old labourer was admitted to the National Hospital, Queen Square, in October 1966, having had a grand mal seizure two years ago and intermittent paresthesiae for the past eight months. There had also been some morning headache and deterioration of memory but he had remained at work. On examination, there was bilateral papilloedema and diminished sensation on the left side of his face. At craniotomy a large, solid left frontal tumour was partially excised. The patient made a good recovery and was alive six months later. Histological examination of the tumour showed " A uniform neoplasm invading both grey and white matter. It consists mainly of loosely packed large cells, with a considerable amount of eosinophilic cytoplasm, separated by loose fibrillar material. Nuclei are darkly staining and pleomorphic. Most of the cells of the neoplasm have one nucleus but a few have two or even three. Mitoses are occasionally present. Other cells have similar nuclei but only a little ill defined cytoplasm. The blood vessels are not unusual in number or calibre, show slight endothelial hyperplasia and slight perivascular cuffing by small mononuclear cells. (Gemistocytic) Astrocytoma grade 3."

*Case* 2.—A 34-year old woman was admitted to Atkinson Morley's Hospital in November 1967 with a four months' history of headache, vomiting and ataxia from the recurrence of a right temporal glioma for which she had had a craniotomy, elsewhere, in March 1965. On examination she had bilateral papilloedema with left facial weakness and a left hemiparesis. At craniotomy a large solid tumour was partially excised. Histological examination of the specimen showed " A widely infiltrating astrocytoma grade 3. It is of gemistocytic type with occasional multinucleated tumour giant-cells."

The tumour specimens were sent to the laboratory and washed with saline to remove adherent blood. Extracts (25% w/v for case 1 and 50% w/v for case 2) were prepared in isotonic saline using a glass tissue homogeniser and the remaining tissue stored at $-15°$ C. Centrifugation for 30 minutes at 3000 r.p.m. and 5° C. provided the required supernatant.

*B. Blood from tumour patients*

*Case* 1.—A blood specimen was taken for serum on the second post-operative day and a heparinised specimen, for red cells, was obtained at a later date.

*Case* 2.—A heparinised sample for red cells and plasma was taken on the first post-operative day.

*C. Specimens for comparison*

*Tissue showing the normal 5-banded pattern.*—Extracts were prepared from. another astrocytoma, Kernohan grade 3, and from a sample of the mixed grey and white matter of a normal frontal pole, amputated as part of another neurosurgical procedure.

*Tissue with a spontaneously occurring supernumerary band.*—Specimens of post-mortem human testis and rat kidney were extracted in the same way as the brain tissues.

*Electrophoresis*

*Agar gel.*—A slightly modified form of the apparatus described by Wieme (1959) was used. The extracts were diluted with 0·1 M phosphate buffer pH 7·4 to give LDH activity of about 2000 i.u. per litre. 5 $\mu$l. of the diluted extract was applied 4·5 cm. from the cathodic end of a standard 2·5 × 7·5 cm. microscope slide, covered with an approximately 1·5 mm. thick layer of 0·8% Difco Noble agar in pH 8·7 barbitone buffer (ionic strength = 0·05). A constant current of 45 mA was applied for 30 minutes, after which time the slides were developed by incubation in the dark for 30 minutes at 37° C. The incubation mixture used was similar to that of Barnett (1964) but made up half strength and in a volume just sufficient to bathe the agar layer of one slide when inverted on two thin strips of Perspex in a petri dish. (0·2 ml. of approximately 0·1 M sodium lactate pH 7·4, 0·2 ml. of 0·5% aqueous nicotinamide adenine dinucleotide, 0·6 ml. of 0·05% aqueous 3-(4,5-dimethylthiazolyl 1–2) 2,5-diphenyl tetrazolium bromide (M.T.T.) and 0·06 ml. of freshly prepared 0·05% aqueous (N-methyl phenazonium methosulphate)).

*Cellulose acetate membrane.*—The buffer system and staining technique of Barnett (1964) was used. The diluted extracts (5 $\mu$l.) were applied 5 cm. from the cathodic ends of the membranes (2·5 × 12 cm.) and separation of the isoenzymes carried out at a constant current of 15 mA for four hours.

*Starch gel.*—Preparation of the starch gel and electrophoresis of the extracts were carried out by a modified form of the method of Smithies (1955). The buffer system consisted of, bridge buffer pH 8·0, 0·3 M boric acid and 0·05 M NaOH and gel buffer pH 8·5, 0·02 M boric acid and 0·008 M NaOH. A gel concentration of 12 g. starch (Connaught hydrolysed starch) per 100 ml. of gel buffer was used. Small pieces of Whatman 3 MM paper were impregnated with the undiluted extracts, and placed in a slit made 6 cm. from the cathodic end of the gel plate (14·0 × 17·5 × 0·6 cm.). A constant current of 20 mA was applied for five hours at 5° C. After slicing of the gel, the isoenzyme patterns were demonstrated by placing sheets of Whatman 1 MM paper on the cut surfaces of the gel, and soaking the paper with the incubation medium of Barnett (1964). The gels were then covered with sheets of polythene and the colour developed by incubation in the dark, at 37 ° C. for one hour.

The extracts used for the cellulose acetate and starch techniques were freshly prepared from the reserves of tissues which had been frozen for periods of a few days (case 2) and for up to six months (case 1, human testis, rat kidney).

*Inhibition studies.*—Inhibition studies on agar gel and cellulose acetate were carried out using extracts prepared from the frozen reserve of tissues. After the method of Ressler, Cook, Olivero and Joseph (1965*a*) 2-mercaptoethanol and *n*-butanol were added to the extracts in varying concentrations up to a final concentration of one part to one part of extract.

*Incomplete incubation mixture.*—Electropherograms of the above extracts were incubated in the absence of NAD and/or PMS (Ressler *et al.*, 1965*a*).

### RESULTS

*Agar gel electrophoresis.*—Fig. 1 shows the pattern obtained from the case 1 tumour extract diluted 1 in 10 and 1 in 20, with the results of similar dilutions from another grade 3 astrocytoma, cerebral hemisphere and testis for comparison. Fig. 2 shows the case 2 tumour extract run at dilutions of 1 in 5 and 1 in 10. In both case 1 and case 2 there are two distinct bands in the position occupied by $LDH_2$ in the typical glioma slide. In case 1 the activity in this position is equally distributed between the two sub-bands, while in case 2 the greater part of the activity lies in the more anodic of the $LDH_2$ sub-bands.

The abnormal bands were detected in tumour extracts stored at $+4°$ C. for two or three days but after a week only the usual 5-banded patterns were obtained. A fresh extract prepared from the frozen reserve of tumour 1, after six months, again showed the abnormal band but no $LDH_5$ was demonstrated. A specimen of tumour 2 has not yet been stored for this period of time. Serum, plasma and red cells of both patients showed only the normal five bands and the distribution of the serum isoenzymes was not abnormal. The testis extract showed a sixth band, migrating between isoenzymes 3 and 4 but on agar the rat kidney showed only the normal 5-banded pattern.

Apart from these two examples supernumerary bands have not been demonstrated in any of the remaining 31 tissue specimens or 50 cyst fluids from astrocytomas grades 3 and 4, or in 68 tissue and 58 cyst fluid samples from other types of cerebral tumour that have been examined in this laboratory.

*Cellulose acetate membrane electrophoresis.*—Electrophoresis on this medium demonstrated the supernumerary bands of the atypical tumour and testis extracts in the same relative positions as on agar gel, but again failed to show an abnormal pattern for the rat kidney extract. Under the conditions employed all the isoenzymes migrated towards the anode.

*Starch gel electrophoresis.*—Fig. 3 shows the patterns obtained from tumour 1, from another grade 3 astrocytoma, human testis, rat kidney and a cerebral secondary carcinoma. All five extracts were run simultaneously on the same starch block to compare the relative positions of the supernumerary bands from different sources on this medium. Tumour 1 (Fig. 3, lines A and D) showed only the anodic migration; as well as the three normal fractions there was the additional band, immediately cathodic to $LDH_2$, in the same relative position as on agar. The 4th and 5th fractions were not demonstrated at the concentration employed in this extract of a stored frozen specimen. The electropherogram, not illustrated, of an extract of tumour 2 presented a similar picture, but isoenzyme 4 was visible. The other astrocytoma showed only the same five bands as it had on agar (Fig. 3, line B) and the five normal isoenzymes of LDH were also seen in the extract from the cerebral secondary (Fig. 3, line F). In contrast to its 5-banded pattern on agar, on starch the rat kidney showed six bands, with the additional band lying immediately anodic to isoenzyme 3 (Fig. 3, line E). An unexpected finding was the appearance of two supernumerary bands in the human testis extract (Fig. 3, line C). Band X lay just anodic to $LDH_4$ as it did on agar, and a fainter additional band was found just cathodic to $LDH_2$, occupying the same relative position as the single supernumerary band of the unusual glioma. $LDH_5$ was not found on the starch electropherogram of the testis extract. To confirm that the second supernumerary band was not a consequence of storage, three fresh post-mortem specimens of human testis were extracted and run in a similar manner and all again showed the two additional bands.

*Inhibition studies.*—No specific resistance to either 2-mercaptoethanol or *n*-butanol was shown by the additional band in the tumour extracts or by the " X " band of the testis extract on agar gel, starch gel and cellulose acetate membrane.

The supernumerary isoenzyme was not demonstrated by the incomplete incubation mixture.

### DISCUSSION

Electrophoresis on starch, agar and polyacrylamide gels and on cellulose acetate membrane separates the LDH of most human tissues and sera into five distinct isoenzymes. These are generally considered to result from a random tetrameric association of two different polypeptide chains which are under separate genetic control and are variously referred to as the heart muscle type, H or B sub-unit and the skeletal muscle type, M or A sub-unit (Appella and Markert, 1961; Cahn, Kaplan, Levine and Zwilling, 1962; Markert, 1963; Shaw and Barto, 1963).

Multiple and single additional bands of LDH however, have been found in extracts of normal tissues from man, rat, mouse and rabbit. The spontaneous occurrence of multiple LDH sub-bands has been reported in human red cells by Boyer, Fainer and Watson-Williams (1963), Nance, Claflin and Smithies (1963) and Vesell (1965) and also by Kraus and Neely (1964) who found a similar pattern in the serum of persons with a variant pattern of their red cell enzyme. All four studies employed starch gel and the sub-bands, from 2 to 15 in number, were attributed to genetic variants involving both the A and B sub-units of the enzyme.

---

### EXPLANATION OF PLATE

FIG. 1.—Agar gel electropherograms of LDH isoenzymes showing the 6-banded patterns of tumour case 1 and human testis together with the 5-banded patterns of an astrocytoma grade 3 and normal cerebral cortex. All slides run under the same conditions. Fastest moving fraction, $LDH_1$, on the left of the figure.

A = Tumour case 1. B = Astrocytoma grade 3.
C = Normal cerebral cortex. D = Human testis.

FIG. 2.—Agar gel electropherogram showing the 6-banded LDH isoenzyme pattern of tumour case 2.

FIG. 3.—Simultaneous starch gel electropherograms showing the relative positions of the supernumerary bands of tumour case 1, human testis and rat kidney, compared with the typical patterns of an astrocytoma grade 3 and a cerebral secondary carcinoma.

A = Tumour case 1. B = Astrocytoma grade 3.
C = Human testis D = Tumour case 1.
E = Rat kidney F = Cerebral secondary carcinoma.

1

2

3

Buckell and Barnes.

These abnormalities were uncommon, occurring in 8 of the 940 persons examined by Kraus and Neely (1964) and in 4 of 1200 in Vesell's (1965) series. Multiple sub-bands have also been found by starch gel electrophoresis in extracts of normal muscle from the mouse and rabbit (Fritz and Jacobson, 1965) and in extracts of rabbit liver run on polyacrylamide gel (Theret and Lalegerie, 1967).

Multiple bands where normally only a single isoenzyme is found, have been produced by chemical manipulation of tissue or tissue extract. Ressler and Tuttle (1966) reported that formaldehyde treatment of post-mortem samples of human testis, kidney, liver and heart, before extraction of the enzyme, resulted in zymograms with two bands in the position of $LDH_2$, three in the position of $LDH_3$, and four in the position of $LDH_4$. At certain concentrations 2-mercapto-ethanol acts in a somewhat similar way.

A single supernumerary band, designated band X, in extracts of human testis has been demonstrated between isoenzyme 3 and 4, at pH 8·6, on starch gel (Blanco and Zinkham, 1963; Ressler, Olivero and Joseph, 1965*b*), on agar gel (Clausen and Ovlisen, 1965) and on polyacrylamide gel (Goldberg, 1963). The X band resisted the inhibitory action of 2-mercaptoethanol and *n*-butanol while the other five bands were completely inhibited (Ressler *et al.*, 1965*b*). No X band was found in pre-pubertal testis or in two post-pubertal specimens showing a marked decrease in spermatogenesis (Blanco and Zinkham, 1963). The relative migration rate of band X was reported by Ressler *et al.*, (1965*b*) to be dependent on the pH of the supporting medium, a change in pH from 8·6 to 7·0 displacing band X from between isoenzymes 3 and 4 to between isoenzymes 4 and 5, but to be independent of whether the supporting medium was starch or agar. The position of the single supernumerary band in extracts of rat kidney as described by Ressler *et al.* (1965*a*) was, however, affected by the supporting medium. At pH 8·6 this band migrated between isoenzymes 2 and 3 on starch gel but in the position of isoenzyme 2 on agar gel. The supernumerary kidney band, like the X band of human testis, showed a selective resistance to inhibition by 2-mercapto-ethanol but, unlike band X, could not be demonstrated with an incubation mixture devoid of PMS and/or NAD.

The LDH of the cerebral tumours described above would appear to have a number of points of interest: Is the supernumerary band an artifact or a real entity? Is this band similar to any of the additional bands described in the literature? Is it related to the nervous system or to cancer?

It seems unlikely that this was an artifact; it was demonstrated in the same relative position on all three of the media employed and was not affected by dilution. It persisted in the deep frozen tissue for at least six months, after which time, as is usual, $LDH_4$ and $LDH_5$ could no longer be detected. The specimen did not come into contact with formaldehyde or any other chemical, nor is the picture like the multiple banded structure described for the chemically produced sub-bands. Glioma tissue does not appear to be susceptible to this type of change; an attempt was made to produce additional bands by treating both tumour tissue and its extract with formaldehyde but no change in isoenzyme pattern was obtained.

On agar, the additional band from the gliomas ran in a different position to the X band of testis (Fig. 1). On starch, the second supernumerary band of testis, not previously described, occupied the same position as the abnormal glioma band (Fig. 3). An attempt to distinguish between testis band X and the glioma band by means of inhibition was unsuccessful as the selective inhibition described by Ressler *et al.* (1965*b*) could not be reproduced; it was not possible therefore to see if the second abnormal position in the testis extracts was running with $LDH_2$ on agar rather than just behind it as on starch.

A remarkably similar picture to line A of Fig. 1 and to Fig. 2 is shown by Soetens, Karcher, van Sande and Lowenthal (1964). These authors describe the occurrence, in 2 out of 18 cerebral tumours, of a single supernumerary fraction of LDH that ran on agar between fractions 2 and 3. This band is not present in normal brain. The distribution of LDH isoenzymes in human brain has been examined by Gerhardt, Clausen, Christensen and Riishende (1963), Gerhardt and Petri (1965), Gerhardt, Clausen, Christensen and Riishende (1967) and in this laboratory, with no mention of the occurrence of anything other than the five normal fractions.

The significance of the appearance of this supernumerary fraction of LDH in relation to malignancy has yet to be determined. It is of interest that all four examples so far described have been in brain tumours. No histological details are given by Soetens *et al.* (1964) but the relative increase in $LDH_5$ shown in the upper of the two slides in their Fig. 3 suggests a malignant tumour. The overall frequency with which this abnormality occurs may not be as high as the 2 out of 18 tumours found by these authors. In this laboratory the supernumerary band has occurred only twice out of 149 patients with cerebral tumours; 33 malignant gliomas, 68 other tumours and 108 cyst fluids have been examined. Gerhardt *et al.* (1963 and 1967) do not report any abnormal bands in their studies of LDH isoenzymes in cerebral tumours.

Supernumerary bands have not been described in the LDH isoenzyme patterns of human neoplastic tissues from organs other than brain. Gibson and Barnett (1963) and Barnett and Gibson (1964) using cellulose acetate examined breast cancer and later extended their work (Gibson and Barnett, 1964) to colon, uterus and bladder. Yasin and Bergel (1965) described the LDH isoenzymes of eight gastric carcinomas on starch gel. Both the foregoing techniques are capable of showing the abnormal glioma band. A further five gastric carcinomas have been examined by Leese (1965) using agar and Baume, Builder, Fenton, Irving and Piper (1966) examined the LDH isoenzyme patterns of seven gastric carcinomas, using cellulose acetate membrane.

It would seem, therefore, that the abnormal fraction of LDH found in these two grade 3 astrocytomas is not an artifact and resembles two examples already described in what was probably a similar type of tumour. It does not occur in the normal nervous system and so far, has not been detected in the limited number of other tumours examined by suitable techniques. The abnormal fraction was confined to the tumours, not being present in the blood of any of the four cases or in post-mortem specimens of lung, kidney, muscle and nerve from one of Soeten *et al.'s* (1964) patients.

### SUMMARY

A sixth band in the agar gel, starch gel and cellulose acetate membrane electropherograms of lactate dehydrogenase (LDH) was found in extracts from 2 of 33 surgical specimens of malignant gliomas. In each case the supernumerary fraction migrated just cathodic to $LDH_2$ and so resembled the only other examples reported of single additional LDH bands in human tumours. This extra band was shown to differ from the X-band of testis and the supernumerary band of rat kidney but migrated, on starch, in the same relative position as a 7th, previously undescribed, band that appeared in the testis extracts. The literature regarding additional LDH bands is reviewed.

This work was carried out with financial support from the British Empire Cancer Campaign for Research and from the Medical Research Council. We thank Mr. Wylie McKissock and Mr. Alan Richardson for providing the tumour specimens and Dr. W. A. Evans and Dr. M. R. Crompton for the histological details of case 1 and 2 respectively.

### REFERENCES

APPELLA, E. AND MARKERT, C. L.—(1961) *Biochem. biophys. Res. Commun.*, **6**, 171.

BARNETT, H.—(1964) *J. clin. Path.*, **17**, 567.

BARNETT, H. AND GIBSON, A.—(1964) *J. clin. Path.*, **17**, 201.

BAUME, P. E., BUILDER, J. E., FENTON, B. H., IRVING, L. G. AND PIPER, D. W.—(1966) *Gastroenterology*, **50**, 781.

BLANCO, A. AND ZINKHAM, W. H.—(1963) *Science, N.Y.*, **139**, 601.

BOYER, S. H., FAINER, D. C. AND WATSON-WILLIAMS, E. J.—(1963) *Science, N.Y.*, **141**, 642.

CAHN, R. D., KAPLAN, N. O., LEVINE, L. AND ZWILLING, E.—(1962) *Science, N.Y.*, **136**, 962.

CLAUSEN, J. AND OVLISEN, B.—(1965) *Biochem. J.*, **97**, 513.

FRITZ, P. J. AND JACOBSON, K. B.—(1965) *Biochemistry, N.Y.*, **4**, 282.

GERHARDT, W., CLAUSEN, J., CHRISTENSEN, E. AND RIISHEDE, J.—(1963) *Acta neurol. scand.*, **39**, 85.—(1967) *J. natn. Cancer Inst.*, **38**, 343.

GERHARDT, W. AND PETRI, C.—(1965) *Acta neurol. scand.*, **41**, 609.

GIBSON, A. AND BARNETT, H.—(1963) *Rep. Br. Emp. Cancer Campn*, **41**, 644.—(1964) *Rep. Br. Emp. Cancer Campn*, **42**, 639.

GOLDBERG, E.—(1963) *Science, N.Y.*, **139**, 602.

KRAUS, A. P. AND NEELY, C. L.—(1964) *Science, N.Y.*, **145**, 595.

LEESE, C. L.—(1965) *Eur. J. Cancer*, **1**, 211.

MARKERT, C. L.—(1963) *Science, N.Y.*, **140**, 1329.

NANCE, W. E., CLAFLIN, A. AND SMITHIES, O.—(1963) *Science, N.Y.*, **142**, 1075.

RESSLER, N., COOK, U., OLIVERO, E. AND JOSEPH, R. R.—(1965*a*) *Nature, Lond.*, **206**, 828.

RESSLER, N., OLIVERO, E. AND JOSEPH, R. R.—(1965*b*) *Nature, Lond.*, **206**, 829.

RESSLER, N. AND TUTTLE, C.—(1966) *Nature, Lond.*, **210**, 1268.

SHAW, C. R. AND BARTO, E.—(1963) *Proc. natn. Acad. Sci. U.S.A.*, **50**, 211.

SMITHIES, O.—(1955) *Biochem. J.*, **61**, 629.

SOETENS, A., KARCHER, D., VAN SANDE, M. AND LOWENTHAL, A.—(1964) In ‘ Enzymes in Clinical Chemistry. ’ Edited by Ruyssen, R. and Vandendriessche, L. Amsterdam (Elsevier), p. 130.

THERET, C. AND LALEGERIE, P.—(1967) *Path. Biol., Paris*, **15**, 14.

VESSEL, E. S.—(1965) *Science, N.Y.*, **148**, 1103.

WIEME, R. J.—(1959) *Clinica chim. Acta*, **4**, 317.

YASIN, R. AND BERGEL, F.—(1965) *Eur. J. Cancer*, **1**, 203.